# How sharing is the “sharing economy”? Evidence from 97 Airbnb markets

**DOI:** 10.1371/journal.pone.0266998

**Published:** 2022-04-21

**Authors:** Petter Törnberg

**Affiliations:** Department of Human Geography, Amsterdam Institute for Social Science Research, University of Amsterdam, Amsterdam, The Netherlands; Institute for Advanced Sustainability Studies, GERMANY

## Abstract

Digital platforms such as Airbnb have become a major economic and political force in recent years, presenting themselves as a “sharing economy”–a new, more just way of organizing social and economic activity–while functioning as owners and managers of proprietary markets. These platforms have in recent years been subject to variegated but growing regulations, begging questions of how these affect their platform markets. This paper examines these claims by a large-scale international comparative analysis of the revenue distribution of Airbnb markets in 97 cities and regions, focusing on the level and evolution of revenue inequality, and estimating the racial and gender revenue gaps by using machine learning classification of host profile pictures. Examining 834,722 listings, 513,785 hosts, and 13,466,854 reviews, the paper finds an average Gini coefficient of 0.68, implying that a majority of the market revenue tends to go to about 10% of the hosts. The level of centralization varies significantly across cities, but is consistently growing over time, with government regulation appearing as a counteracting factor, which however only temporarily slows down the growing dominance of a small minority of large-scale hosts. The paper furthermore finds large gender and race revenue gaps, as Black hosts receive on average 22% less revenue for their listings, and women an average of 12% less. These findings contribute important data to ongoing academic and policy debates, as well as a starting point for further research on inequality in the sharing economy, and how it can be regulated.

## Introduction

The quickly growing “sharing economy” platforms promise an alternative to the market and the state–a form of social organization that is disintermediated and without central leadership, but yet freed from the tendencies toward economic inequality associated to the free market [1–4]. Short-term rental platforms like Airbnb are an important part of this “sharing economy”, referring to themselves as part of a growing “movement” for enabling a marketplace of small-scale exchange, using terms like “home sharing” or “peer-to-peer” [5]. Platforms like Airbnb can be understood as owners and rent-seekers of proprietary markets, which link private real-estate capital with short-term renters. By lowering the thresholds of participation in the short-term rental economy, Airbnb claims to support a flexible way for lower and middle-income communities to “make ends meet”–said to particularly benefit women and minorities [6]. These claims of positive community impact are often interwoven with a larger story in which they are said to be shaping a path toward a new economy, enabling more egalitarian outcomes through a basis in more convivial forms of exchange.

The claim that these platforms enable more “sharing” and egalitarian forms of exchange to take place within their bounds are not fundamentally implausible: digital technology has shown significant capacity to shape social life, nudging users toward certain behavior while dissuading others, and even organize social spheres based on non-commercial exchange [7]. One does not have to look as far as Wikipedia to find examples of sharing in digital platforms [1]: CouchSurfing, to which short-term rental platforms historical roots can be traced, was indeed based on ideals of sharing and non-monetary forms of exchange, as hosts did not charge but opened their homes for visitors in the hopes that others would do the same [8]. While having brought in a monetary component, platforms like Airbnb describe themselves as part of this lineage.

Critics, however, argue that these stories about home sharing are mere “sharewashing”–a thin façade that helps platforms rally popular political support for bypassing the regulations of traditional tourism accommodation [9]. These critics suggest that while Airbnb is indeed moving tourism accommodation into low and middle-income minority communities–in part by bypassing zoning regulation–this means that the costs, not the benefits, are accrued by these communities [10,11], while the profits flow to large-scale finance capital [12]. These counter-points are embedded in their own larger narrative, with activists and scholars describing platforms not as bringing about a “sharing economy”, but as harbingers of a new era of privatization and laissez-faire economy that Srnicek [13] refers to as “platform capitalism”. With growing criticism from scholars and affected citizens, platforms like Airbnb have in recent years become subject to variegated but growing regulations, with cities imposing new taxes and limitations to short-term rental. These regulations are aimed in part at stemming the tendencies toward large-scale real estate capital using the platforms to bypass regulation on hotel industry, and to push the platforms towards use as small-scale rentals.

This background begs the question of *who* the users are that are operating on and benefitting from Airbnb are–large-scale operations or small-scale private individuals, men or women, ethnic minorities or majorities–and whether the implemented regulations are efficacious in shifting the platform markets towards smaller-scale use. Existing studies have tended to focus on one or two of a small set of cities in the Global North, with limited studies looking at Airbnb markets comparatively, internationally, or longitudinally, which has limited the possibility to examine the effects of context and regulation on the centralization and inequalities of their proprietary markets. This paper contributes to this debate by asking: (1) To what extent is the Airbnb marketplaces dominated by small-scale rental, rather than large-scale professional operations? and (2) How large are the race and gender revenue gaps in the Airbnb marketplaces? The answers to these questions provide important clues to the question of whether Airbnb is small-scale rental platform, or whether it rather appears to reproduce, perpetuate, or even exacerbate existing economic inequalities in terms of class, race, and gender. As the study is international and comparative, it speaks to questions of how and whether local regulation and context shape the structure of platform markets, which has grown in importance as governments have begun to increasingly regulate the platform economy. To answer these questions, the paper uses data from 97 cities and regions to quantitatively examine the revenue distribution of the marketplaces.

### The home sharing revolution

Short-term rental platforms such as Airbnb are the successors of non-profit platforms such as CouchSurfing [8], on which hosts did not charge but opened their homes for visitors in the hopes that others would do the same for them. However, as venture capital started to move into the sector in search for profitable investments, the platforms shifted toward monetization and short-term rentals [13,14]. These short-term rental platforms have seen explosive growth in recent years, with Airbnb, the largest and most iconic of the platforms, claiming 150 million users world-wide, and a total of half a billion stays in their 6 million listings (as per late 2020). While Airbnb was the first large-scale short-term rental platform, many established travel companies have in recent years begun moving into the market. These platforms have come to fundamentally change how people travel, challenging the traditional hotel industry.

Airbnb claims to enable small-scale “home sharing”, by lowering thresholds for participation on the short-term rental market, and thereby providing an extra income to low-income families and communities. In this narrative, the platforms stand on the side of the regular homeowner against large-scale real-estate interests, by supplanting large-scale hotel industry with small-scale home rental [15–19]. Airbnb has also released multiple reports promoting this narrative, such as a 2016 report suggesting that Airbnb helps middle-class minority families “make ends meet” by allowing them to rent out spare sofas and extra rooms [20]. Airbnb purports that it helps make everyone a “small-scale entrepreneur”, by providing an open and level playing-field, widening participation in the short-term rental market to underprivileged groups [5]. The suggestion is, in short, that Airbnb’s marketplace is characterized by small-scale rentals, and that it is relatively egalitarian in terms of traditionally underprivileged groups.

This “home sharing” idea is in turn related to the broader notion of a “sharing economy”, founded in the affordances of new digital technology to mobilize new forms of social organization [2]. Platform technology has breathed new life in the utopian visions of societies organized without both central political authority and economic inequalities–pointing to Wikipedia as illustrating evidence, and its capacity to channel the unpaid efforts of hundreds of thousands of volunteers to collectively building a vast and constantly evolving repository of human knowledge [1]. The idea that digital technology can enable people to harmoniously self-organize, promoting smaller scale and more egalitarian marketplaces has animated much of the enthusiasm for “sharing economy” platforms [3,13].

Critics of Airbnb, on the other hand, argue that the narrative of “sharing” is nothing but a guile of the platforms real function: to provide a vehicle for real estate capital to by-pass regulation and taxation. In this narrative, Airbnb is not on the side “regular homeowners” against real-estate interests, but rather serves precisely the latter, by enabling a new asset class for investments for global financial capital [12]. These critics suggest that what makes Airbnb competitive with hotels in many cities is precisely the ability to flaunt zoning regulations, allowing it to expand the hospitality industry into residentially zoned areas, and avoid taxation to thereby offer cut-rate prices. Airbnb uses various means to do so, including claiming that responsibility to pay taxes and fulfill legal requirements are carried by its hosts–while simultaneously refusing to share host information, suing governments and tax agencies, and wielding their user community as a lobbying power to fight stringent regulation [16,21,22].

The debate about Airbnb has intensified as concerns over the platform’s impact on cities and communities have grown, with residents raising complaints against unsustainable levels of urban tourism in residential areas brought about by these platforms [23]. As protests against Airbnb are becoming more frequent, some governments are beginning to respond by regulative measures, such as requiring permits, fees, taxation, putting a cap on the number of days an apartment can be rented–or simply prohibiting short-term rental altogether [21]. There has however been limited studies on how these regulations are in practice affecting the platform’s proprietary markets, and on whether they are successful in stemming large-scale rental operations. Most studies have focused on single cities, predominately in the so-called Global North, which has limited the possibility to speak to the debate on the efficacy of different forms of regulation.

Central to the debate on the regulatory treatment of Airbnb is thus the empirical question of how “sharing” the Airbnb marketplace actually is. This is the question towards which we now turn, by examining inequalities in a large number of Airbnb markets of different sizes from around the world.

## Method and data

This study takes a heterodox approach to studying Airbnb, using digital data for the critical examination of the societal implications of the platform [24]. Accessing the data of platforms such as Airbnb is challenging as the company does not share their data, implying that studies are forced to rely on web-scraped data. The analysis is carried out using data from InsideAirbnb [25]. InsideAirbnb is a noncommercial database based on scraping all available data from the Airbnb interface at regular and specified intervals, including Airbnb listings, their geographic location, information on hosts, all reviews of the listings, price per night, and much more, for a specified geographic region. The data source provides useful basis for studying Airbnb, and has been used by a large number of research studies [26–33]. In this study, current data for all available regions were collected, as well as all historic data, consisting of data scrapings collected by InsideAirbnb in previous years [25]. InsideAirbnb collects data on the most important Airbnb markets, which includes major cities and tourist destinations, as well as cities and regions for which particular research interest has been reported [see 25].

Since the study focuses on measuring revenue inequality, a relative measure of the revenue of a host is needed. Since Airbnb does not make public data on host revenue, research [e.g., 34,35] and government reports [36] on Airbnb uses the number of reviews and the price of listings, together with an approximated average booking duration and an estimated probability of guests posting a review, to approximate host revenue. There has been some discussion regarding the average booking duration and review probability, however, for calculating the level of inequality, we do not in fact need an approximation of revenue, but simply a value that is *proportional* to the revenue. To calculate such a value, we follow the standard practice of multiplying the number of reviews over the given time period with the price charged (Airbnb presents prices converted to the currency of the user, and InsideAirbnb collects listing prices in USD), and sum this up for each listing of the host. Again, this does not constitute an estimation of the revenue, but what we refer to as the *relative* revenue: a value proportional to the revenue acquired. Like any proxy for estimating the share of host revenue from available data, this has some important limitations and is based on assumptions, which will be discussed below. Hosts with no reviews are not included in the revenue calculations.

The study has three empirical parts: (1) the current level of inequality; (2) the evolution of inequality; (3) race and gender revenue gaps.

To examine the current level of inequality (1), we focus on reviews made during 2019 (since 2020 was an unrepresentative year in tourism due to the COVID-19 pandemic.) The data for 2019 was collected by acquiring the first InsideAirbnb scraping following the end of 2019, that is, in January 2020, for each city. These data were used to calculate revenues for 2019, which in turn were used to estimate the inequality by identifying the probability distribution function of the revenue distribution, calculate the Lorenz curve, and Gini coefficient of all available cities.

For to examine the evolution of inequality (2), the same calculations were made over time, using data from previous data collections by InsideAirbnb. These data, however, are only available for a selection of cities. The nine cities for which historic data with regular collection going back to 2015 were selected. Each time-period is treated as the time between one data dump and the next, using the latest information on listings and reviews to calculate the revenues. These values are then used to calculate separate Gini coefficients, revealing change over time.

Finally, to examine race and gender revenue gaps (3), we determine gender and race of hosts using the face analysis machine learning API Clarifai to automatically classify profile pictures. Pictures with no identifiable face (these constituted 26%) or multiple faces (these constituted 22%) were discarded. Clarifai uses the categories Black, East Asian, Indian, Latino-Hispanic, Middle Eastern, Southeast Asian, and White. The model returns a value between 0 and 1 for each racial category. The highest value was used to represent race as a nominal variable. Using these values, the impact of race and gender was then estimated using an OLS regression model. The dependent variable (revenue) is first normalized by dividing it by the average and multiplying it by 100. The variable thus represents the host revenue compared to the average revenue in percentages, meaning that the coefficients of the OLS will describe the difference in percentages in relation to the mean revenue. It should be noted that as this is an exploratory analysis, which does not have explanative aims, quality of fit measure (such as R^2^), are not relevant for the evaluation of the model and will not be presented in the analysis.

To validate the accuracy of the image classification model, we used a name-to-gender API to identify the gender of all hosts. These data were however not employed directly in the model, as (1) such APIs have been shown to be biased by ethnicity [37], which makes them inappropriate for the purpose at hand, and (2) data on only gender without race do not allow separating the effects of these variables. These data do, however, allow further validating the accuracy of the image classification. Using the Genderize API, 91.5% of hosts were successfully classified using their first names. Comparing these data to the image classification results showed that 92% of hosts that were classified as women by the name-to-gender API were also classified as women by the image classification. This verifies the high accuracy of the image classification.

It should be noted that the aim of this paper is not to explain inequalities on the platform, but to measure the size of the gender and ethnic revenue gaps. These revenue gaps will be the result of a combination between direct discrimination (such as guests choosing hosts on the basis of their gender or skin-color), and indirect discrimination (such as institutional racism, and pre-existing racial inequities in housing capital). While much Airbnb research has focused on isolating the effect of direct discrimination, this paper argues that institutional and structural inequalities should not be “controlled away”, as the most important impact of Airbnb in terms of inequality arguably is providing the means to convert existing (cultural and economic) capital to new revenue. The term “revenue gap” is used in order to emphasize that the aim is to *descriptively capture the headline level of income difference between the groups*, as opposed to the explanatory aim of identifying the causal effects of direct discrimination on host revenue.

The data were collected and analyzed using custom-written code in Python, using standard packages such as Pandas, Seaborn and StatsModels [38], running on a Jupyter Lab running on Google Cloud Platform.

A number of limitations with the approach should be noted. First, the estimation of relative revenue is likely to have some biases, in particular in that there may be small differences in the likelihood that a particular host receives a review. For instance, a guest may be more prone to submit a review when hosted by a personal and small-scale host, than by a large anonymous company, which would suggest that the calculated inequality is a low estimate. Similar differences in review probability or average stay length may also exist in between genders and ethnic groups, which may skew the results. While some studies instead employ proprietary algorithms from companies such as AirDNA to approximate occupancy rate using data on listing availability, this does not necessarily resolve these biases, but does render them impossible to assess, as the company has not made public the details of its algorithm [39]. A second limitation is the assumption that the host profile features a photo of the actual host. This may not always be the case, for instance, hosts may attempt to avoid discrimination by using a photo of someone else. Rental companies may use their logo instead of a face, which means that some large hosts may not be included in the gender and race gap calculation. However, these remain the best estimates available, and similar limitations apply to much of this empirical literature.

The author would furthermore note the challenges relating to the use and operationalization of the notion of “race” as used here, in particular in comparison with self-described racial categories. As an operationalization of this elusive phenomenon, automatic identification of skin color has obvious and important limitations, as race is a contested and socially constructed category, and thus far from merely a question of skin color [40]. Similar challenges apply to the notion of gender, which here, for reasons of methodological constraints, is treated as a binary. In the case of both gender and skin color, the profile picture representation does however have the benefit of capturing race as it is seen by other users on the platform. The approach taken here furthermore provides us with a necessary departure point, from which we can have at least a tentative look into the racial and gender inequities of the global sharing economy.

To ensure anonymity for hosts and guests, all data were anonymized, and no data on individuals were stored. The profile images used in the analysis were not saved beyond the use for machine learning classification, and the resulting data cannot be traced back to individual users.

## Results

**Fig 1:**
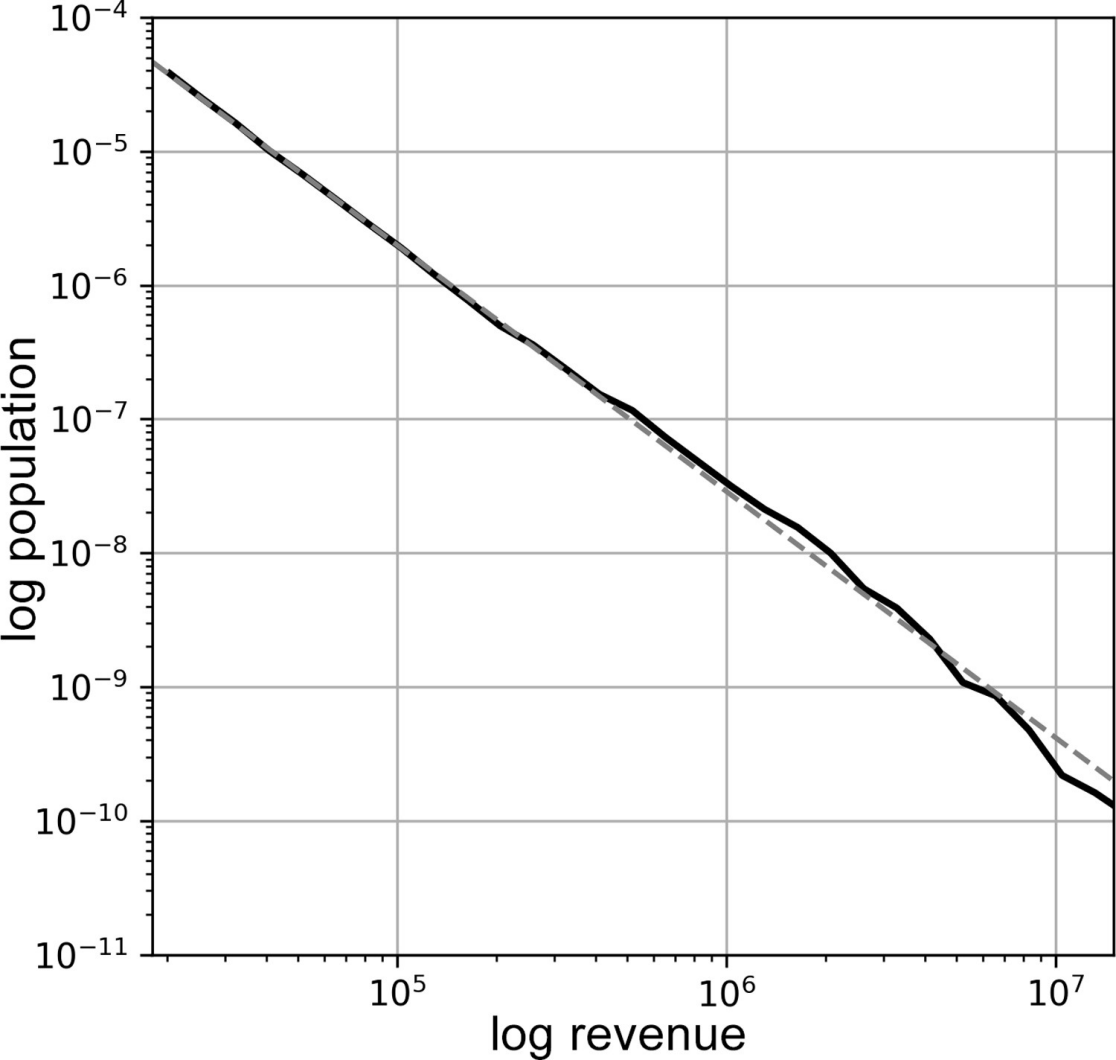
A log-log plot of the probability distribution function of the revenues of the 456,489 hosts in all 97 cities, showing the distribution of revenue over the population of hosts. This is fit with a power law distribution (dashed line), with α = 1.840. This analysis was carried out using *powerlaw* [41] based on the approach suggested in Clauset et al [42].

To get a first preliminary overview, we begin by examining the distribution of host revenue over the entire population, that is, including all 456,489 hosts of the 97 cities in the data during 2019. As Fig 1 shows, the host revenue approximates a power-law distribution, expressed as a straight line on a log-log plot. Power-law distributions are highly unequal, here indicating that an exponentially small number of hosts are receiving the bulk of the revenue. The shape of the distribution already provides an important clue to the level of inequality on Airbnb, as these distributions are commonly the result of feedback mechanisms–such as the attention-drawing-attention dynamics on digital platforms, in which certain messages spread exponentially, while others remain largely unseen. This may suggest that a similar feedback dynamic may be at play in the distribution of rental revenue on Airbnb, in which revenue results in additional revenue, as profitable listings become more visible on the platform.

**Fig 2:**
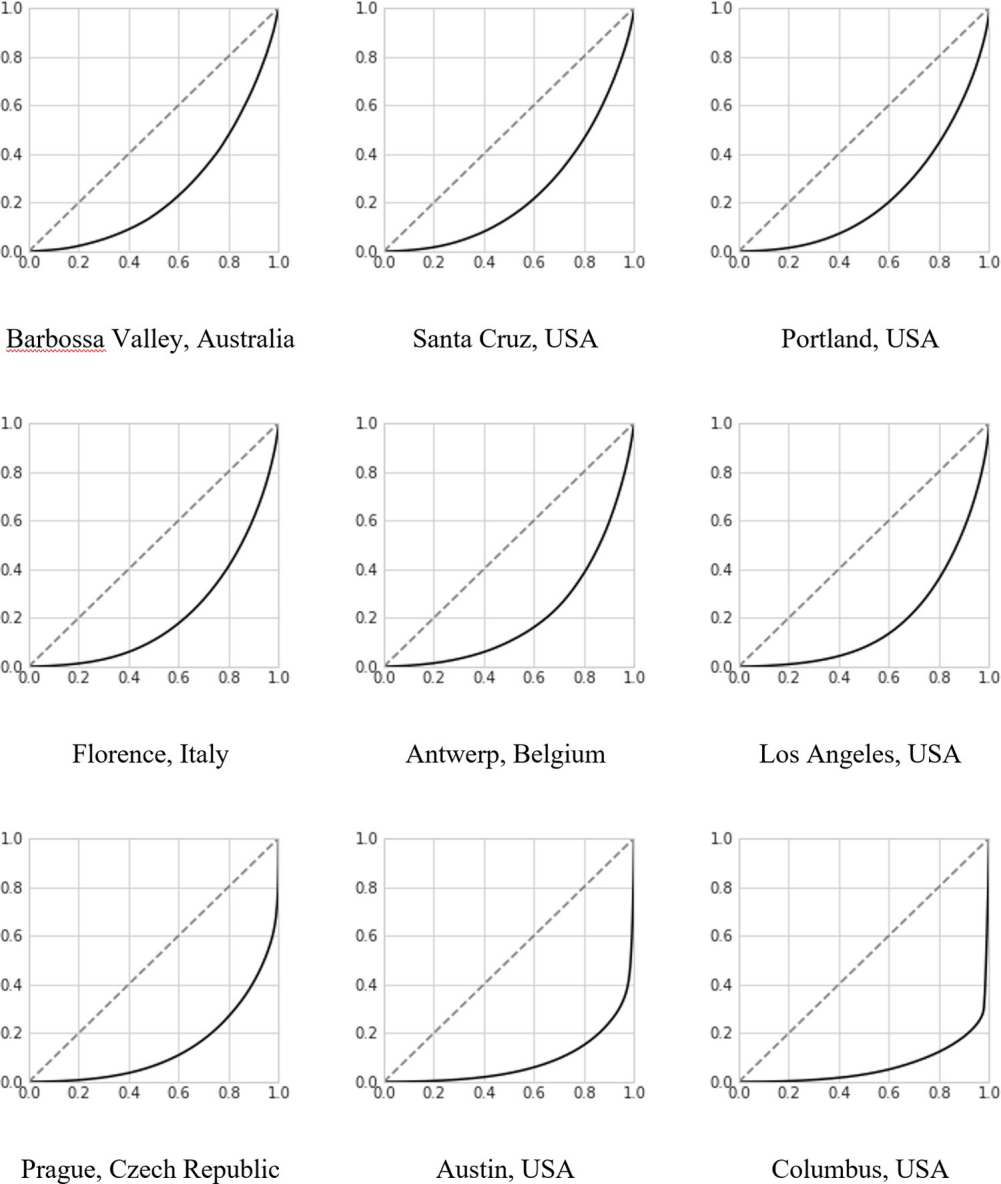
The Lorenz curves for the three cities with lowest, median and highest Gini coefficients, respectively. The Lorenz curve shows the cumulative fraction of the population on the x-axis, and the cumulative fraction of the revenue taken by this population on the y-axis. For instance, looking at Prague, that the line is at y = 0.3 at x = 0.8 tells us that 80% of the hosts receive around 30% of the revenue. The dashed line shows the distribution that corresponds to everyone taking an equal share. (Gini is defined as the area between the straight and the curved line divided by the entire area under the straight line).

To compare and identify the level of revenue inequality in the different cities, we turn to calculating the Gini coefficient for each of the 97 cities. The Gini coefficient is a common measure of inequality, defined using the Lorenz curve (see Fig 2) as the area between the diagonal line and the curve, divided by the entire area below the diagonal line. Table 1 shows the Gini coefficients, as well as the number of listings in the city, the number of hosts that manage these listings, the number of total reviews, as well as the average reviews per listing, and average listings per hosts. Together, these measures give a sense of the state of the rental market in the city at hand.

**Table 1:** The number of listings, hosts, reviews, and Gini coefficients for 2019 of the 97 cities included.

Country	Market	Listings	Hosts	Reviews	L./H.	R./L.	Gini
**Australia**	Barossa Valley	211	149	3,277	1.42	15.53	0.49
**United States**	Santa Cruz county	1,346	943	34,997	1.43	26.00	0.54
**United States**	Portland	3,880	3,072	126,058	1.26	32.49	0.55
**Canada**	Victoria	2,971	2,208	71,560	1.35	24.09	0.57
**Australia**	Tasmania	4,611	3,122	112,638	1.48	24.43	0.57
**United States**	Asheville	2,121	1,519	68,430	1.40	32.26	0.58
**Canada**	Vancouver	4,820	3,506	98,454	1.37	20.43	0.58
**Canada**	New Brunswick	1,847	1,229	35,264	1.50	19.09	0.59
**Australia**	Western Australia	9,868	6,326	157,446	1.56	15.96	0.61
**Spain**	Menorca	1,669	964	10,083	1.73	6.04	0.61
**Spain**	Girona	9,768	5,466	67,900	1.79	6.95	0.61
**United States**	Rhode Island	2,935	1,906	47,762	1.54	16.27	0.61
**United States**	Salem OR	**138**	110	3,547	1.25	25.70	0.62
**Italy**	Venice	7,153	3,462	174,302	2.07	24.37	0.62
**Greece**	Thessaloniki	2,316	1,581	42,745	1.46	18.46	0.62
**Belgium**	Ghent	996	746	24,348	1.34	24.45	0.62
**Australia**	Barwon South West Vic	4,461	2,751	81,005	1.62	18.16	0.62
**United States**	Denver	3,717	2,777	106,377	1.34	28.62	0.62
**Denmark**	Copenhagen	14,535	13,444	116,207	**1.08**	7.99	0.63
**Spain**	Mallorca	9,085	3,778	75,329	2.40	8.29	0.63
**United States**	Cambridge	869	434	20,378	2.00	23.45	0.63
**Italy**	Naples	5,272	3,520	92,560	1.50	17.56	0.63
**United States**	Seattle	5,767	3,415	144,051	1.69	24.98	0.63
**Greece**	Crete	10,394	6,086	73,570	1.71	7.08	0.63
**United States**	Oakland	2,134	1,444	42,732	1.48	20.02	0.63
**France**	Bordeaux	6,247	5,199	86,038	1.20	13.77	0.63
**Spain**	Valencia	5,687	3,513	110,683	1.62	19.46	0.64
**Switzerland**	Vaud	2,512	1,921	25,838	1.31	10.29	0.64
**United States**	San Francisco	5,750	3,493	228,563	1.65	39.75	0.64
**United States**	Hawaii	17,016	7,126	262,528	2.39	15.43	0.64
**United States**	New York City	30,187	22,293	480,131	1.35	15.91	0.64
**Italy**	Bologna	3,374	2,199	63,752	1.53	18.90	0.64
**United States**	New Orleans	5,860	3,339	140,268	1.76	23.94	0.65
**Spain**	Malaga	4,747	2,276	85,524	2.09	18.02	0.65
**United States**	Washington Dc	5,661	3,604	135,758	1.57	23.98	0.65
**Italy**	Bergamo	1,509	1,053	21,879	1.43	14.50	0.65
**The Netherlands**	Amsterdam	12,030	10,582	140,681	1.14	11.69	0.66
**Norway**	Oslo	4,163	3,632	49,965	1.15	12.00	0.66
**Australia**	Northern Rivers	4,486	2,813	66,894	1.59	14.91	0.66
**Canada**	Quebec City	2,245	1,520	57,062	1.48	25.42	0.66
**United States**	Pacific Grove	169	112	5,387	1.51	31.88	0.67
**United Kingdom**	Bristol	2,003	1,305	41,829	1.53	20.88	0.67
**Italy**	Puglia	15,491	10,586	87,969	1.46	**5.68**	0.67
**Portugal**	Porto	8,905	4,510	191,881	1.97	21.55	0.67
**Italy**	Sicily	25,157	16,982	204,262	1.48	8.12	0.67
**Germany**	Munich	5,959	5,074	60,382	1.17	10.13	0.67
**Italy**	Florence	8,965	4,828	204,933	1.86	22.86	0.67
**Belgium**	Antwerp	1,674	1,125	30,698	1.49	18.34	0.68
**United States**	Los Angeles	26,625	15,310	532,650	1.74	20.01	0.68
**Portugal**	Lisbon	18,408	8,638	364,078	2.13	19.78	0.68
**Belize**	Belize	1,757	663	16,505	2.65	9.39	0.68
**United States**	Nashville	6,144	3,095	174,491	1.99	28.40	0.68
**Switzerland**	Geneva	2,019	1,491	26,960	1.35	13.35	0.68
**France**	Lyon	6,240	5,228	84,976	1.19	13.62	0.68
**United States**	Twin Cities MSA	3,501	2,221	67,834	1.58	19.38	0.69
**Italy**	Rome	21,586	12,102	441,336	1.78	20.45	0.69
**United States**	Santa Clara County	5,791	2,805	93,272	2.06	16.11	0.69
**United Kingdom**	Greater Manchester	3,563	2,105	63,923	1.69	17.94	0.69
**Brazil**	Rio De Janeiro	14,468	10,172	122,638	1.42	8.48	0.70
**Sweden**	Stockholm	3,664	3,193	41,110	1.15	11.22	0.70
**Canada**	Ottawa	2,442	1,516	53,666	1.61	21.98	0.70
**Spain**	Sevilla	5,538	2,641	142,884	2.10	25.80	0.70
**Greece**	Athens	7,925	4,613	173,732	1.72	21.92	0.70
**Taiwan**	Taipei	7,222	2,297	150,821	3.14	20.88	0.71
**United States**	Boston	2,604	1,034	58,663	2.52	22.53	0.71
**Spain**	Euskadi	3,934	2,521	72,599	1.56	18.45	0.71
**France**	Paris	38,100	31,611	470,971	1.21	12.36	0.71
**United States**	Jersey City	2,261	912	43,465	2.48	19.22	0.71
**Austria**	Vienna	9,173	5,553	190,395	1.65	20.76	0.72
**Canada**	Toronto	15,524	9,621	292,774	1.61	18.86	0.72
**Greece**	South Aegean	14,425	7,881	112,891	1.83	7.83	0.72
**Argentina**	Buenos Aires	13,970	9,507	162,636	1.47	11.64	0.72
**United States**	Chicago	6,861	3,831	156,245	1.79	22.77	0.72
**United States**	Broward County	7,334	3,551	108,462	2.07	14.79	0.73
**United Kingdom**	London	**50,697**	30,300	583,316	1.67	11.51	0.73
**Italy**	Milan	12,155	8,390	199,251	1.45	16.39	0.73
**China**	Beijing	21,854	9,072	200,697	2.41	9.18	0.73
**Belgium**	Brussels	5,841	3,862	114,469	1.51	19.60	0.73
**Ireland**	Dublin	5,661	3,762	260,107	1.50	**45.95**	0.73
**Australia**	Melbourne	16,526	9,391	265,617	1.76	16.07	0.74
**South Africa**	Cape Town	13,072	8,585	129,054	1.52	9.87	0.74
**United States**	San Diego	9,659	5,009	210,366	1.93	21.78	0.74
**United Kingdom**	Edinburgh	9,102	6,622	204,913	1.37	22.51	0.75
**Germany**	Berlin	13,817	11,200	195,295	1.23	14.13	0.75
**Mexico**	Mexico City	14,450	8,552	242,512	1.69	16.78	0.76
**Spain**	Madrid	15,078	8,367	319,775	1.80	21.21	0.76
**Australia**	Sydney	20,161	13,445	251,739	1.50	12.49	0.76
**China**	Hong Kong	5,944	2,713	76,216	2.19	12.82	0.77
**Japan**	Tokyo	12,072	3,323	205,638	**3.63**	17.03	0.77
**United States**	Clark County Nv	7,225	3,379	137,955	2.14	19.09	0.77
**Singapore**	Singapore	3,764	1,099	42,753	3.42	11.36	0.78
**Canada**	Montreal	11,863	7,414	196,517	1.60	16.57	0.79
**Spain**	Barcelona	13,980	7,136	273,465	1.96	19.56	0.79
**Turkey**	Istanbul	8,926	5,115	91,018	1.75	10.20	0.80
**Czech Republic**	Prague	10,727	4,833	256,109	2.22	23.88	0.82
**United States**	Austin	7,194	4,470	141,126	1.61	19.62	0.89
**United States**	Columbus	1,194	591	35,044	2.02	29.35	0.92

Average Gini: 0.68. Median Gini: 0.68. Std. dev: 0.069. Highest and lowest values highlighted in bold.

Two things stand out in Table 1. First, the large variance in many of the indicators between different cities and regions. The number of reviews per listings, for instance, ranges from Dublin, Ireland, with 45.9 reviews/listing to Puglia, Italy, with 5.68 reviews/listing. That listings in Dublin see such a large number of guests implies that these listings are primarily used for rental, and that they are seeing high occupancy. Similarly, the number of listings per host vary from 1.08 in Copenhagen, Denmark, to 3.63 in Tokyo, Japan, indicating the extent to which the market is dominated by professional hosts with a large number of listings. The second take-away from the Table 1 is the overall high level of inequality on the platform. Fig 2 reveals a more fine-grained way of examining the revenue inequality: the so-called Lorenz curve, which shows the cumulative fraction of the population on the x-axis, and the cumulative fraction of the revenue taken by this population on the y-axis. This allows seeing what fraction of the population represents what the fraction of the total revenue.

The average Gini coefficient for the included cities is 0.68, with 10% of hosts representing roughly 50% of the market revenue. In the cities with the highest levels of inequality, such as Columbus (Ohio, USA), and Austin (Texas, USA), and Prague (Czech Republic), however, a large share of the market revenue is going to just 1% of the hosts.

The level of inequality does not seem to be strongly determined by the country in which the city is located: United States has the cities with the highest level of inequality (Columbus, Ohio and Austin, Texas) but also has some of the cities with the lowest inequality (Asheville, North Carolina). This is not completely surprising, since the regulation so far to large degree has taken place on the municipal, state, or city-level. There does seem to be some relationship to the level of regulation in different cities, as the cities with the lowest level of inequality have relatively strong regulations against Airbnb. For instance, Santa Cruz County requires a permit for short-term rentals and puts a cap on the number of allowed days. Portland similarly requires a short-term permit, and does not allow the rental of secondary residence like a second home or vacation rentals. Asheville has also enacted increasingly strict regulations starting in 2015, culminating in 2018 with a ban on nearly all types of short-term rentals, with fines of $500 per day. Vancouver also only allows short-term rental of one’s principal residence, in other words, where you live most of the year and the residential address you use for bills, identification, taxes, and insurance. It should be noted that while the cities that disallow secondary home rental tend to have a lower number of listings per host, they are all well above 1, implying less than full compliance.

At the other end of the list, showing the most unequal markets, we find Columbus, Ohio. Ohio is one of the states with the least regulation of short-term rentals, and while Columbus began requiring permits, insurance and tax-payment from Airbnb hosts in 2018, they are lenient towards non-owner-occupied rentals and there is no cap on the number of days that investors can rent out their properties on Airbnb. Austin, Texas has similarly lax policies toward Airbnb, as does Prague, Czech Republic, requiring only payment of basic taxes and fees.

There are however exceptions to this pattern of strong regulation leading to lower inequality. Singapore has virtually prohibited Airbnb, with hosts risking $20,000 fines and eviction. Japan has similarly strict policies, removing 80% of the listings in 2018. Despite of this, both have a relatively high Gini index. A possible reason is that their markets are relatively small, and that most host may be professional hotels using the platform to advertise their properties.

### Evolution of inequality

**Fig 3:**
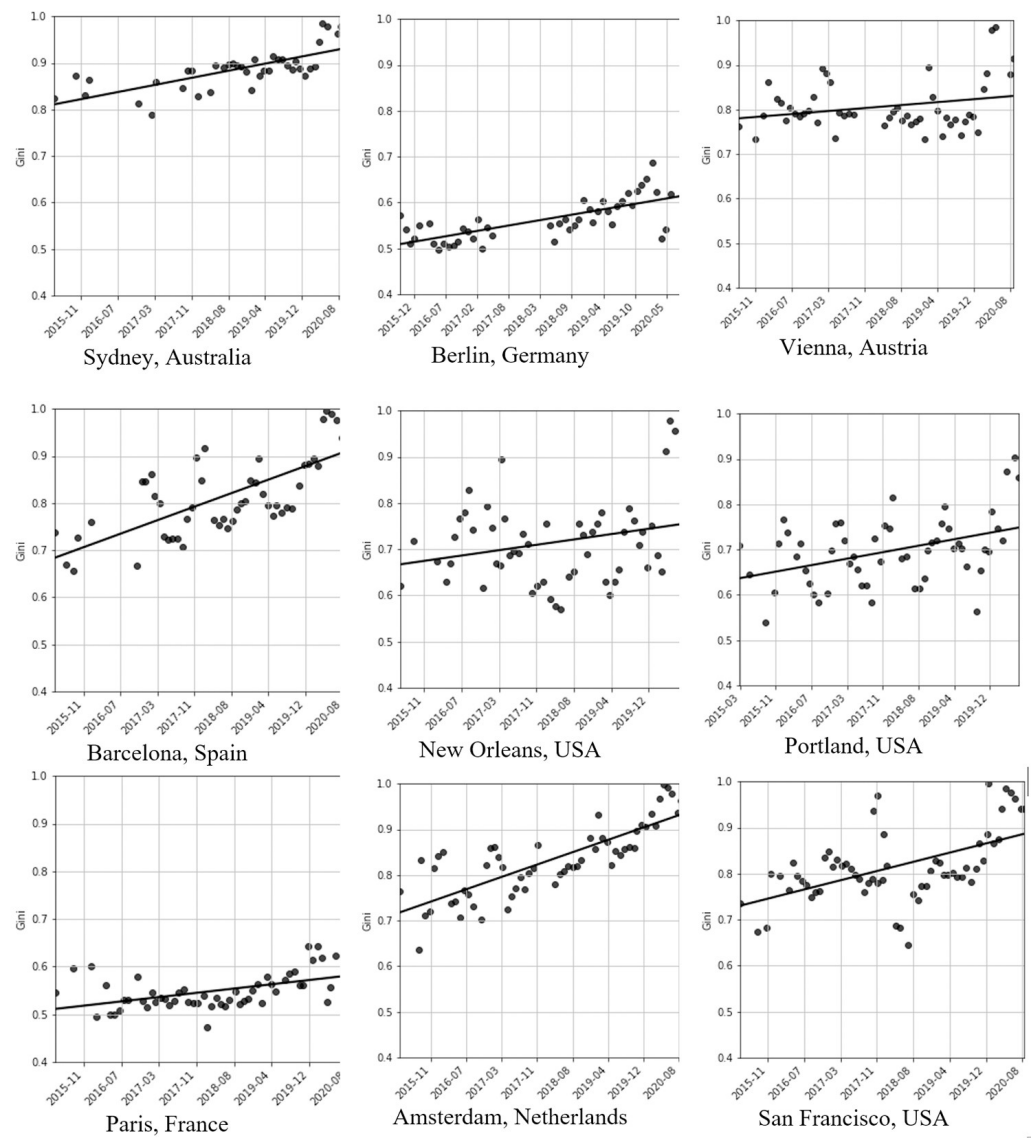
The evolution of the Gini coefficient measured for the nine cities for which data is available since 2015. A OLS tendency line is included to show the evolution of the measure.

To examine the evolution of inequality on Airbnb, we focus on the nine cities for which regular data is available since 2015. Fig 3 shows the resulting figures (it should be noted that the inequality for these shorter periods may differ from the full-year inequality, since longer periods means that more hosts will have non-zero revenue.) As the figures show, some cities, such as Portland or Barcelona, have significant variability of the coefficient with the seasonal flux in visitors, while other cities, such as Paris, are less seasonal and remain relatively stable throughout the year. Strikingly, all cities show an upward trend of the Gini coefficient, implying that the markets are becoming more unequal over time.

Surprisingly, while cities with more regulation tend to have lower levels of inequality, regulation against Airbnb does not significantly seem to impact the long-term evolution of the Gini coefficient. Amsterdam, for instance, instituted a 30-day annual cap and tourist tax on January 1^st^, 2019. This did not impact the city’s trend toward a centralization of its market. San Francisco struck a deal with Airbnb in 2018, resulting in the automatic registration of Airbnb hosts in the city. While registering had been required since 2015, enforcement had been challenging without the collaboration of Airbnb. This change caused almost half the listings to be removed from the platform. The results can be clearly seen in the graph, showing a quick spike in inequality, followed by a corresponding drop, before returning to its initial trajectory of growing inequality. This suggests that the platform has an inherent tendency toward growing inequality, which can be reduced but not completely counteracted through policy action.

### Race and gender gaps

We now turn to examining the revenue gaps between demographic groups. We here focus on the 14 cities with the most hosts, as our resources for automatic classification were limited, and focusing on the cities with most host maximized statistical power.

We begin by looking at the percentage of hosts of each city of each ethnic appearance category (see Table 2). While comparing self-reported racial group with the group identified by image analysis classification is somewhat perilous, it remains notable that the Black population on Airbnb is significantly lower than the population of the cities. For instance, while 25.1% of the population of New York City is Black according to official demographics, only 14.3% of the hosts on Airbnb are classified so. Similarly, in Rio de Janeiro, 11.5% of the population identify as Black (*preto*) and 36.5% colored (*pardo*), while only 3.6% of the hosts on Airbnb are classified as Black. In London, 13.3% of the population is Black, but only 7.5% of hosts are classified as Black. In Cape Town, 38.6% describe themselves as “Black” and 42.4% as “Colored”, and only 1.4% as White. On Airbnb, in contrast, only 4.8% are classified as Black, and 74.4% are classified as White–constituting an over 5,300% overrepresentation of White hosts. This overrepresentation is likely driven in part by racial segregation–with privileged populations tending to be overrepresented in urban areas attractive to tourism–as well as by White hosts being more likely to invest in rental real-estate in minority-majority tourism areas [see 11].

**Table 2:** Fraction of identified hosts of each majority ethnic group and gender appearance in the included cities, focusing on data for 2019.

	Black	East Asian	Indian	Latino-Hispanic	Middle Eastern	Southeast Asian	White	Female	Male
**London**	7.5%	4.9%	3.3%	6.5%	17.1%	1.7%	59.0%	53.3%	46.7%
**Paris**	2.8%	4.0%	1.1%	5.8%	17.6%	0.8%	67.9%	51.9%	48.1%
**New York**	14.3%	8.9%	2.8%	9.0%	15.8%	2.3%	46.8%	53.1%	46.9%
**Beijing**	1.4%	74.3%	0.6%	1.8%	4.2%	3.0%	14.7%	65.3%	34.7%
**Rome**	2.2%	3.0%	1.7%	6.1%	24.9%	0.9%	61.2%	51.1%	48.9%
**Sydney**	2.3%	16.7%	1.9%	5.5%	14.3%	3.1%	56.2%	57.7%	42.3%
**Copenhagen**	1.6%	2.2%	0.7%	3.6%	9.5%	0.8%	81.6%	59.1%	40.9%
**Rio de Janeiro**	3.6%	2.3%	2.0%	14.5%	22.5%	0.8%	54.3%	55.4%	44.6%
**Berlin**	2.7%	3.9%	1.2%	4.9%	14.2%	1.1%	72.0%	49.7%	50.3%
**Buenos Aires**	1.6%	2.3%	1.1%	7.0%	25.5%	0.7%	61.6%	53.5%	46.5%
**Toronto**	5.4%	17.9%	4.1%	7.9%	17.0%	4.0%	43.6%	54.0%	46.0%
**Melbourne**	1.9%	26.0%	1.9%	5.0%	10.5%	3.2%	51.4%	57.0%	43.0%
**Amsterdam**	2.9%	2.6%	0.9%	4.6%	11.3%	0.9%	76.8%	52.1%	47.9%
**Cape Town**	4.8%	1.9%	1.0%	5.6%	11.2%	0.8%	74.7%	58.5%	41.5%
**ALL**	4.6%	12.5%	1.9%	6.3%	15.7%	1.7%	57.2%	54.9%	45.1%

A consistent underrepresentation can be found in relation to male hosts. Table 2 shows that in all cities except Berlin, women are overrepresented as hosts. In Beijing, only 34.7% of hosts are classified as male, and in Copenhagen the number is 40.9%. A possible explanation is that that the home and the associated reproductive labor tend to fall under the domain of the woman, which would imply that women carry out the work associated to these rentals.

Turning to the question of revenue gaps, we run a separate OLS regression model for each city to calculate the size of the differences between demographic groups in the cities. Table 3 summarizes the regression coefficients for the included cities, using White males as the baseline, and normalizing the coefficients to show difference compared to the mean in percentages.

**Table 3:** The ± column show 0.95 confidence interval.

	Black	±	Latino	±	E.Asian	±	M.Eastern	±	Indian	±	SE.Asian	±	Female	±	Nr
**London**	-39.2***	4.2	-23.3***	4.5	-17.4***	5.1	-8.6***	3	-18.7***	6.1	-16.1*	8.4	-16.9***	2	31,901
**Paris**	-27.8***	10	0.5	7.4	-1.5	8.7	-4.0	4.6	-21.5	16	16.6	19	-15.1***	4	21,030
**New York**	-11.3***	3.6	-10.4**	4.4	-14.1***	4.4	-6.5*	3.5	-11.7	7.4	-2.4	8.2	-11.5***	2	17,978
**Beijing**	-31.7**	15	-41.3***	13	-31.3***	4.7	-26.6***	9.1	33.4	21	-3.3	11	9.9***	4	15,639
**Rome**	7.1	9.4	-2.7	5.8	-7.2	8.1	-3.5	3.3	-4.4	11	22.7	14	-5.8**	3	13,022
**Sydney**	-22.3	19	4.8	13	3.7	8	17.0**	8.7	-26.5	21	-6.1	17	-6.6	6	11,800
**Copenhagen**	-25.8*	14	-3.8	9	9.4	11	8.5	5.8	-25.4	21	5.6	20	-24.2***	4	11,110
**Rio de Janeiro**	-47.1***	11	-23.3***	6	-25.3*	14	-19.4***	5.2	-29.0**	15	-24.1	23	-32.4***	4	10,925
**Berlin**	-29.4**	13	2.1	9.5	-13.6	11	-17.3***	6	-44.4**	19	31.1	20	-22.2***	4	10,099
**Buenos Aires**	-3.6	21	5.5	10	-14.3	17	1.5	6.2	-24.0	24	-15.0	31	-8.3	5	9770
**Toronto**	-16.8	16	2.0	14	-7.0	10	-5.4	10	117.8***	19	-14.1	19	-4.0	7	9418
**Melbourne**	13.7	13	-21.6***	8	-11.9***	4.1	-1.3	5.8	-40.5***	13	-25.1***	9.8	-0.6	4	8914
**Amsterdam**	29.3***	10	14.9*	8	27.6***	11	-5.2	5.4	-13.2	18	58.1***	17	-19.9***	3	8184
**Cape Town**	-57.9***	13	-6.0	12	-9.2	20	-6.3	8.7	13.1	27	-51.1*	31	-12.4**	6	7652
**ALL**	-22.2***	2.4	-8.9***	1.6	-8.0***	1.6	-4.9***	1.5	-1.9	3.7	-3.4	3.9	-12.2***	1	187,442

* p < .1

** p < .05

***p < .01.

The coefficients for separate OLS models run for each city, and one model running over all data. Baseline is White and male. Revenue is normalized by city average so that coefficient shows difference compared to mean in percentages. Table 4 in Appendix A includes interaction effects, to show intersectionality of skin color and gender.

Focusing on women, we see that the table points to a significant cost to being a woman in most of these cities. In every city except Beijing, female hosts receive less revenue than male hosts. Rio de Janeiro provides the most extreme case of a gender revenue gap, with women receiving 32.4% less revenue.

Focusing on racial groups, we find that in most cities, non-White hosts are disadvantaged. In London, for instance, all racial groups are disadvantaged compared to White hosts, with Black hosts seeing 39.2% lower revenue compared to White hosts. Black hosts are disadvantaged in all cities where the difference is statistically significant, except in Amsterdam. This disadvantage appears larger in cities with larger Black populations. In Cape Town, Black hosts receive 57.9% less revenue, and in Rio de Janeiro, 47.1% less.

The relative privilege of different groups varies significantly between cities–which is to be expected due to the different contexts and histories. Some racial groups see relative privilege in some cities, and strong disadvantage in others. Indian appearance, for instance, is correlated with a strong disadvantage in Melbourne, while being associated to higher revenue in Toronto. Amsterdam overall provides an interesting counterexample to the trends, where both Black, Hispanic-Latino, Southeastern Asian and East Asian hosts receive significantly more revenue than White hosts.

## Conclusion

This paper has used empirical data from 97 cities and regions to examine how much “sharing” can be found in the Airbnb marketplaces, focusing on the level of small-scale rentals, and the revenue gaps between races and genders.

In examining the level of inequality in the Airbnb marketplaces, the paper found that the markets are highly unequal, and that the inequality has increased over time. This suggests that the platform is dominated by a small number of professional hosts, and becoming more and more centralized and professionalized over time. While the Airbnb markets may have started relatively equal, they have since gradually moved toward a small fraction of the hosts taking the lion’s share of the market revenue. Just as the distribution of likes and retweets on social media platforms, the Airbnb revenues are power-law distributed, implying that a Matthew effect is in play: hosts with more revenue being more likely to attain further revenue. This resonates with the findings of Deboosere et al [43], who show that professional hosts tend to attract more revenue. Bosma [44] provide that a possible explanation, suggesting that Airbnb is actively promoting professionalization of the platform by providing additional tools and support for large-scale hosts. These findings suggest not only that Airbnb is not promoting small-scale sharing, but that they are in fact actively pushing for professionalization of the tourist accommodation on the platform. Reductions of inequality and against professionalization in the rental markets appear to come not from the much-vaunted “self-regulation” but rather from forceful regulations and policing from cities and governments. However, these policies are merely slowing down the inexorable march toward centralization, with markets becoming increasingly dominated by a small minority of large-scale hosts.

The paper furthermore found that Airbnb host demographics poorly represent the overall city demographics: Black hosts are often underrepresented, while White hosts are overrepresented as hosts–up to 5300% of the urban demographics, in the case of Cape Town. This overrepresentation is likely in part driven by racial segregation, with White residents being overrepresented in areas that are attractive to tourism–however, this explanation only accounts for real estate which is not primarily used for short-term rentals. As research on Airbnb in New York City has shown, White hosts are strongly overrepresented also in attractive Black-majority neighborhoods, as those who invest in housing property intended for short-term rental tend to be predominantly White [11]. While Airbnb may focus on how their platform benefits minority families, this shows how the groups profiting from the platform tend to be already privileged. While minorities may not be the beneficiaries of the platform’s upsides, they are the main victims of its downsides–most notably rent increase and gentrification [11,45]. Women tend to be overrepresented as hosts. One explanation for this may be that household labor traditionally is seen as the domain of women, meaning that when a household rents out a house on Airbnb, it is the woman who carries out the work involved, such as managing contact with guests, cleaning, etc.

Turning to the question of revenue gaps, the paper found large gaps between genders and racial groups. While women are overrepresented as hosts, they receive less revenue than men in almost all cities. For racial groups, the size and direction of these gaps vary significantly between cities (which is to be expected since, for instance, majority and immigrant status of the groups vary between countries.) Black hosts appear to be the most underprivileged group, in particular in cities and countries with large Black populations–such as Cape Town or Rio de Janeiro, where Black hosts have a 57.9% and 47.1% revenue gap, respectively.

These revenue gaps are likely to have their origins in multiple factors; discrimination in guests choosing host on the basis of their profile picture [27,46–48], having limited access to housing wealth that is attractive to touristic consumption due to e.g. racial segregation [35,49], biases in the machine-learning algorithms operating on the platform [39], and access to the “right” cultural capital to frame one’s neighborhood for touristic consumption [11]. As Airbnb becomes more professionalized, this puts more demand on hosts to have the necessary resources to offer professional tourist accommodation, which further pushes minority and low-income groups out of the market.

The aim of this paper has not been to explain or disentangle the plural mechanisms through which these inequities operate–but to examine the sharing narrative by providing a descriptive account of the level of inequality on the platform. The paper finds that the platform does not dissuade but perpetuates or even exacerbates existing inequalities: Airbnb provides a pathway for those who own housing wealth, who have valued gender and racial appearance, and who own cultural capital, to combine these resources to gain further economic benefit. If, in summary, the “sharing” in the “sharing economy” is taken to imply small-scale rentals and relative equity between races and gender, it can now firmly be said that there is little sharing in Airbnb: the marketplaces are dominated by a small fraction of professional hosts–and are becoming more and more so.

If this is the case, and Airbnb is even actively promoting professionalization, how should we understand their continued focus on the “home sharing” narrative? One possibility is that the narrative itself has become central to the Airbnb business model. Platforms have been allowed to operate in a regulatory vacuum that many critics argue is more significant to their success than any of their technological innovations. As governments are looking to regulate platforms, the ability to wield political influence to shape these regulations is thus becoming increasingly central to the platforms’ business models [50]. This can be seen in how Airbnb is actively engaging in politics to maintain their advantages, leveraging its significant political power to push local governments, organizing “self-organized” social movements in cities around the world to support its interests [22]. This repositions Airbnb, making it less of an “innovative tech firm”, and more of a “regulatory entrepreneur”: its innovation and competitive advantage lie in its ability to support large-scale real estate capital to bypass, fight and litigate public regulations [50]. The “home sharing” narrative can be understood as an important part of the toolkit used to mobilize broad political influence, for a company engaged in a form of technology-driven policy activism.

This blurring of the boundary between technology and politics is thus central to how these platforms should be understood. The lesson from CouchSurfing should not be that there is something inherently “sharing” or “peer-to-peer” about new digital technology, nor should the lesson for Airbnb be that there is something inherently neoliberal about it. Both should tell us that digital technology has powerful capacity to embody institutional functions. If these powers are wielded by venture capital, there is little reason to expect anything but a continuation of their interests–which are, as it seems, the pursuit of “a nightmarish form of neoliberal capitalism”, in Martin’s [51] colorful phrasing. The growing political capacities of technology must be met by corresponding politicization of technological design, if we are to maintain the role of political life as the foundation of governance. While a sharing economy may be possible, it should, in other words, not be expected to appear *deus ex machina*, but only as the product of deliberate political action.

## Appendix A

**Table:** 

	London	Paris	New York	Beijing	Rome	Sydney	Copenhagen
**Black**	-38.2***	-34.8***	-5.8	-37.0**	-9.8	-20.4	-34.5*
**±**	6.1	13.5	5.4	17.7	12.1	24.8	18.9
**Fem. Black**	-1.9	18.7	-10.1	12.0	42.0**	-5.2	18.4
**±**	8.4	21	7.3	34.2	19.3	39.8	27.4
**Latino**	-20.9***	28.3**	-15.9**	-65.4***	6.6	-16.1	7.0
**±**	6.8	11.5	7.1	18.8	9.1	20.2	15.7
**Fem. Latino**	-4.2	-47.1***	8.3	47.4*	-15.6	35.6	-16.1
**±**	9	15	9	26.4	11.8	26.2	19.2
**E.Asian**	-12.7	-2.6	-18.4***	-36.5***	1.2	-4.4	19.1
**±**	8.1	15.1	6.9	8	11.6	13.3	21.1
**Fem. E.Asian**	-7.6	1.0	6.8	7.8	-17.1	12.6	-13.8
**±**	10.4	18.5	9	9.9	16.3	16.7	25.1
**M.Eastern**	-9.6**	0.5	-2.3	-26.0*	-10.4**	26.0**	10.3
**±**	3.9	5.9	4.7	13.3	4.3	11.3	7.7
**Fem. M.Eastern**	3.3	-10.9	-9.8	-3.5	17.2**	-26.1	-4.3
**±**	6.2	9.5	7.2	18.3	6.8	17.8	11.8
**Indian**	-16.9**	-20.7	8.2	-1.3	-33.4***	-30.4	-25.4
**±**	7.6	19.6	10.4	34.5	12.8	27.8	27
**Fem. Indian**	-4.9	0.5	-41.1***	54.5	89.2***	9.4	0.0
**±**	12.9	35.1	14.9	43.4	22.7	43.1	42.8
**SE.Asian**	-17.0	-1.1	17.1	11.6	41.9*	-1.1	-53.2
**±**	15.4	31.4	14.3	15.4	21.4	25.1	38.1
**Fem. SE.Asian**	1.2	27.6	-29.3*	-31.3	-34.7	-9.1	79.7*
**±**	18.4	39.5	17.4	21.1	28.7	33.6	44.4
**Female**	-16.5***	-11.4***	-8.1**	3.9	-10.3***	-6.8	-23.7***
**±**	2.9	4.1	3.5	9	3.5	8	3.8

**Table 4:** To examine the effect of intersectionality of race and gender, we employ a version of the model which includes interaction effects between skin color and gender.

	Rio de Janeiro	Berlin	Buenos Aires	Toronto	Melbourne	Amsterdam	Cape Town
**Black**	-76.9***	-34.8**	10.1		-12.5	48.3***	-54.0***
**±**	16.6	16.1	23.9	22.5	17.1	12.7	18.2
**Fem. Black**	52.7**	13.6	-43.5	-14.8	60.4**	-51.0**	-8.1
**±**	22.2	25.8	46.7	32.5	25.4	20.9	25.2
**Latino**	-28.9***	15.2	19.5	12.4	-23.1*	16.4	-17.5
**±**	9.9	15.1	15.6	21.2	12.3	13	21.6
**Fem. Latino**	9.2	-21.4	-24.3	-18.2	2.8	-2.7	16.7
**±**	12.4	19.5	20.8	27.9	16.2	16.6	25.7
**E.Asian**	-25.0	-40.5**	21.8	-10.7	0.7	46.9***	0.9
**±**	20.4	16.3	27.1	16	6.5	16.7	32.3
**Fem. E.Asian**	-1.4	46.2**	-61.2*	6.6	-21.0**	-32.7	-15.9
**±**	27.3	21.5	35.4	20.5	8.4	21.7	40.7
**M.Eastern**	-27.7***	-16.1**	9.8	-8.9	-2.6	-4.9	-17.6
**±**	7	7.5	8.4	13.8	7.6	6.9	11.4
**Fem. M.Eastern**	17.1	-4.1	-17.2	2.8	6.6	0.1	26.7
**±**	10.5	12.4	12.5	20.9	11.9	11	17.6
**Indian**	-46.5**	-47.6**	-25.3	-3.0	-38.0**	-15.9	21.4
**±**	19.4	22.5	28.2	23.7	15.3	21.6	38.3
**Fem. Indian**	37.5	10.1	16.7	317.2***	-3.2	9.6	-16.9
**±**	29.4	40.6	56.8	37.9	27.4	36.9	53.4
**SE.Asian**	-27.8	27.4	-55.9	-15.2	-6.7	74.2***	-82.5
**±**	36.2	30.7	49.4	29.1	14.4	28.3	54
**Fem. SE.Asian**	5.9	6.3	66.3	2.1	-33.4*	-26.2	46.9
**±**	47.2	40	63.1	37.8	19.6	35.9	65.8
**Female**	-40.3***	-23.0***	-0.8	-16.2	4.1	-17.4***	-15.7**
**±**	5.7	4.8	6.7	11	4.9	3.8	6.3

The resulting interaction effects are complex–in certain places positive and in other places negative: Being Black and woman is, for instance, associated to higher revenue than being Black and male in Rio de Janeiro and Melbourne, but less revenue Amsterdam. The effects are however often not statistically significant, making them more challenging to interpret.
